# Burden of Neurodevelopmental Disorders in Kenyan Children

**DOI:** 10.1001/jamanetworkopen.2025.48853

**Published:** 2025-12-12

**Authors:** Symon M. Kariuki, Patricia Kipkemoi, Martha Z. Kombe, Mary A. Bitta, Jacqueline Phillips Owen, Amina Abubakar, Charles R. J. C. Newton

**Affiliations:** 1 Neuroscience Unit, Kenya Medical Research Institute (KEMRI)–Wellcome Trust Research Programme, Kilifi, Kenya; 2 Department of Public Health, Pwani University, Kilifi, Kenya; 3 African Population and Health Research Center, Nairobi, Kenya; 4 Department of Psychiatry, University of Oxford, Oxford, United Kingdom; 5 Institute for Human Development, Aga Khan University, Nairobi, Kenya; 6 Brain and Mind Institute, Aga Khan University, Nairobi, Kenya; 7 Institute of Psychiatry, Psychology and Neuroscience, King’s College London, London, United Kingdom; 8 South London and the Maudsley NHS Foundation Trust, London, United Kingdom

## Abstract

**Importance:**

Neurodevelopmental disorders (NDDs) such as autism spectrum disorder (ASD), attention-deficit/hyperactivity disorder (ADHD), and intellectual disability contribute to one-tenth of the global burden of mental and neurologic disorders. The burden of NDDs is not well documented in Africa.

**Objective:**

To assess the prevalence, risk factors, and comorbidities of NDDs in young Kenyan children.

**Design, Setting, and Participants:**

This cross-sectional study was conducted among children aged 6 to 9 years residing in the Kilifi Health and Demographic Surveillance System in Kenya. In stage I of the study, children were screened for NDDs from March 16, 2015, to September 14, 2016; those with a positive screening result and a randomly selected proportion of those with a negative screening result were invited to undergo clinical evaluation to confirm the diagnosis (stage II).

**Exposures:**

Adverse perinatal events, medical and family history, and socioeconomic status.

**Main Outcomes and Measures:**

Prevalence of NDDs was estimated, and risk factors and medical comorbidities associated with NDDs were assessed using generalized linear models and link functions.

**Results:**

Of the 11 223 children screened in stage I, 5646 (50.3%) were males, and mean (SD) age was 7.6 (0.96) years. A total of 522 children had an NDD confirmed in stage II, with an overall adjusted prevalence of 9.1% (90.8 [95% CI, 83.4-98.6] cases per 1000 children), which differed by sex (237 of 5577 females [4.2%] vs 285 of 5646 males [5.0%]; *P* = .045). The NDDs with the highest prevalence were ADHD (50.8 [95% CI, 45.2-57.1] cases per 1000 children) and cognitive impairment (27.1 [95% CI, 22.9-31.9] cases per 1000 children); ASD was also common (15.6 [95% CI, 12.6-19.1] cases per 1000 children). Co-occurrence of NDDs was reported in 118 children (22.6%; 95% CI, 19.2%-26.5%). Key factors associated with occurrence or onset of NDDs included adverse perinatal events (adjusted risk ratio [ARR], 1.64; 95% CI, 1.29-2.09), febrile infections (ARR, 1.28; 95% CI, 1.05-1.56), eating soil (ARR, 1.45; 95% CI, 1.13-1.85), snoring (ARR, 1.50; 95% CI, 1.27-1.76), and being landless (ARR, 1.32; 95% CI, 1.12-1.56). Head injuries were unique to developmental conditions and febrile infections to neurologic conditions, with many risk factors shared. Common medical comorbidities for NDDs after adjusting for attrition and screening specificity included motor neuron dysfunction (ARR, 2.79; 95% CI, 2.18-3.56) and malnutrition (ARR, 1.80; 95% CI, 1.37-2.39).

**Conclusions and Relevance:**

NDDs were common in school-aged children in this rural area of Kenya, and the findings suggest their occurrence was associated with preventable environmental risk factors. These disorders and associated comorbidities should be identified and addressed in children in Africa.

## Key Points

**Question:**

What are the extent of and factors associated with neurodevelopmental disorders (NDDs) in young children living in a defined area in Kilifi, Kenya?

**Findings:**

In this cross-sectional study including 11 223 children aged 6 to 9 years, NDDs occurred in 9%, and behavioral manifestations (eg, attention-deficit/hyperactivity disorder) were more common than neurologic manifestations (eg, epilepsy). NDDs were associated with factors including perinatal complications, febrile infections, and medical problems such as malnutrition.

**Meaning:**

The findings suggest interventions and studies should be initiated to understand and address factors associated with NDDs to support parents of affected children and increase understanding among stakeholders.

## Teaser

This cross-sectional study investigates the prevalence of and factors associated with neurodevelopmental disorders in children aged 6 to 9 years in Kenya.

## Introduction

Children have 10% of the global burden of mental and neurologic disorders, most of which have increased since the 1990s.^1^ Childhood mental and neurologic disorders include neurodevelopmental disorders (NDDs), which comprise a range of conditions, including autism spectrum disorder (ASD), attention-deficit/hyperactivity disorder (ADHD), intellectual disability, impairments in hearing and vision, and speech and language disorders. These spectra of disorders are thought to be related because they are all characterized by brain dysfunction during development and share similar genetic and environmental risk factors. Even when conditions such as hearing impairments have a nonneural origin, a neurodevelopmental origin may be considered if brain plasticity ensues.^2^ Grand challenges in global mental health^3^ and the United Nations General Assembly^4^ have recognized the enormous medical and socioeconomic impact of NDDs on children, families, and communities and have urged prioritization of these conditions for identification and management.

The prevalence of NDDs in children is 5% to 10%, based on estimates from high-come countries,^5^ but these estimates may be greater in low- and middle-income countries (LMICs), where many perinatal, infectious, and environmental risk factors are more frequent. A systematic review^6^ showed that the burden of NDDs in LMICs is grossly underestimated because of a lack of studies on NDDs such as ASD and ADHD. This review also found that there was no single study examining all NDDs together in a community except for neurologic impairments such as epilepsy and intellectual disability, with a prevalence lower than that of ADHD. This situation is improving, with an important study of NDDs in India^7^ estimating a prevalence of 9.2% to 13.6% across heterogeneous districts in the country. This study was based on a modest sample, and the population used as the denominator was from a census whose vital statistics are not routinely updated. Due to wide variations in demographics, socioeconomic status, risk factors, and health care systems in LMICs, it is important to carry out more studies in these settings to compare their burden of NDDs and comorbidities with that in other settings worldwide.

The precise burden of NDDs is unknown in Africa, which hinders planning of services by policy makers. Africa has had the greatest increase in burden of NDDs in children younger than 5 years (by 71.3%) of any continent in the world.^8^ A high prevalence of mental health problems (13%) was reported in preschool children from rural areas of Africa,^9^ but this study did not examine the entire spectrum of NDDs, particularly in older school-aged children. Several factors may influence the burden of NDDs in Africa; sometimes, individuals with underlying genetic susceptibility require exposure to additional environmental factors for the onset of an NDD to occur, consistent with the double- and triple-hit hypothesis.^10^ Neonatal and infant mortality decreased by 53% in sub-Saharan Africa from 1990 to 2015^11^; however, children exposed to neonatal insults may survive with neurologic sequelae and neurodisabilities.^4^ In rural areas of Africa, perinatal complications, head injuries, infections, and environmental toxins are common and are associated with onset of developmental problems in young children.^12^ Older children with NDDs experience other medical comorbidities, but to our knowledge there are no population-based studies to provide empirical evidence on this subject in Africa.

Conducting large and high-quality epidemiologic studies of mental and neurologic disorders is now possible in resource-limited settings following improvement in research infrastructure,^13,14^ development of locally appropriate neuropsychological and mental health assessment tools,^15,16^ and training of African research leaders. We therefore conducted an epidemiologic study of school-aged children living in a rural area of Kenya to estimate the prevalence of NDDs and associated risk factors and medical comorbidities.

## Methods

### Ethical Approval

Permission to conduct this cross-sectional study was obtained from the scientific and ethics review unit of the Kenya Medical Research Institute. Parents gave written informed consent for their child to participate. The study followed the Strengthening the Reporting of Observational Studies in Epidemiology (STROBE) reporting guideline.

### Study Site and Population

This study was conducted in a defined area, the Kilifi Health and Demographic Surveillance System (KHDSS), which is located on the Kenyan coast 60 km north of Mombasa. The main population in the area is the Mijikenda ethnic group, most of whom are subsistence farmers and a few of whom are fishers. The literacy level in the area is low, and Kilifi County is among the poorest administrative regions in Kenya. During the period of the study, there was 1 epilepsy and neurodevelopmental clinic, run by neuroscience researchers in collaboration with Kilifi County Hospital.

### Sampling

**Figure 1.**
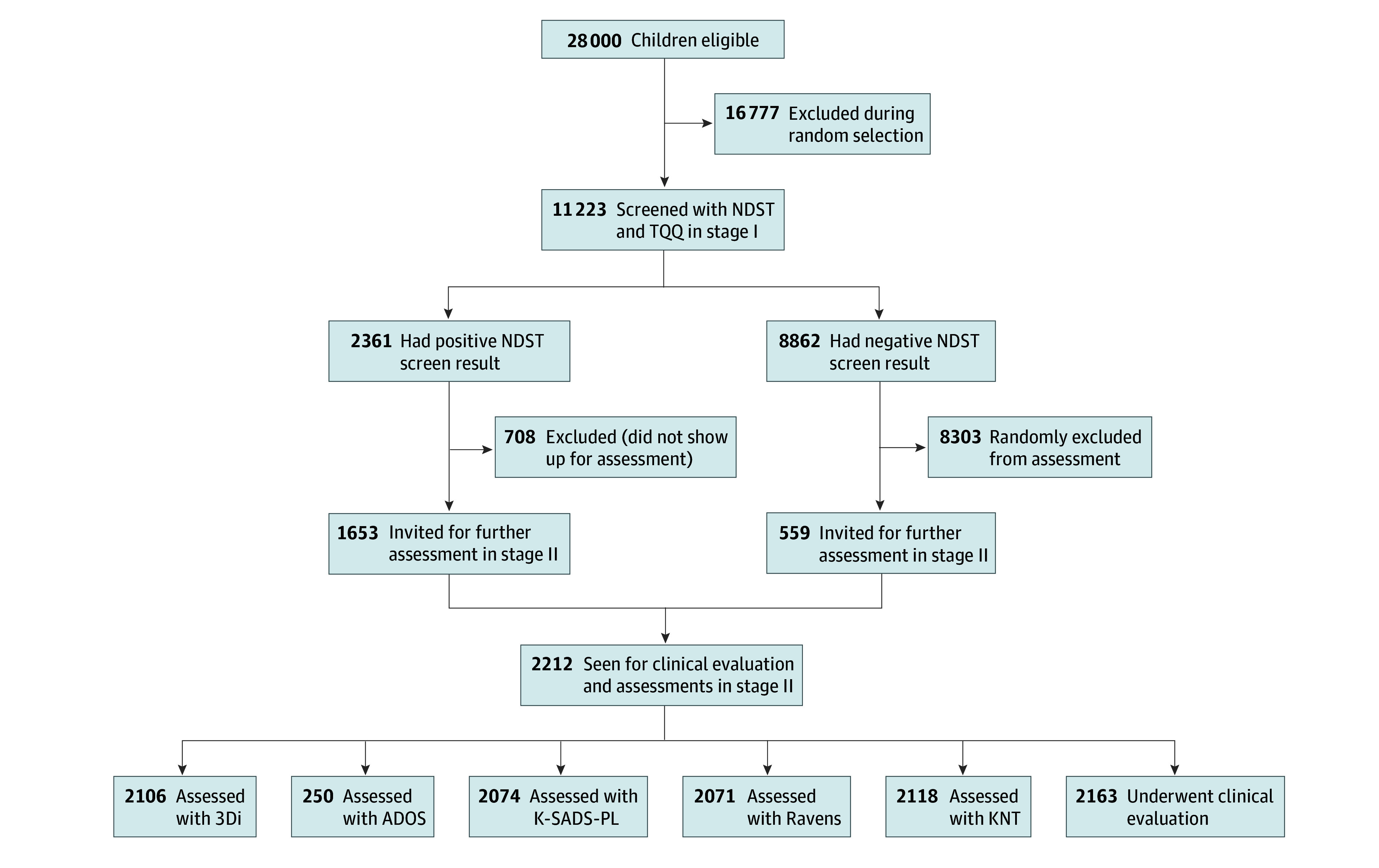
Derivation of the Study Sample Among Children Aged 6 to 9 Years Living in the Kilifi Health and Demographic Surveillance System Area of Kenya Screening for neurodevelopmental disorders was done in stage I, and children with a positive screen result and a randomly selected proportion of those with a negative screen result were invited for detailed clinical evaluation to confirm the diagnosis in stage II. 3Di indicates Developmental Diagnostic Dimensional Interview; ADOS, Autism Diagnostic Observation Schedule; KNT, Kilifi Naming Test; K-SADS-PL, Kiddie Schedule for Affective Disorders and Schizophrenia for School-Age Children–Present and Lifetime; NDST, Neurodevelopmental Screening Tool; Ravens, Ravens Colored Progressive Matrix Test; TQQ, 10 Questions Questionnaire.

This study involved children aged 6 to 9 years living within the KHDSS, who form a total population of about 28 000 (Figure 1). The age range of 6 to 9 years was chosen because most children with an NDD who were exposed to early neonatal and early-life risk factors will have developed the NDD (eg, ADHD) by this age, the sensitivity of detecting these disorders is optimal at this age, and this is the age when children start formal education in Kenya. We estimated that screening of about 11 000 randomly selected participants from the 28 000 children would detect NDDs with a precision or margin of error of less than 1%, assuming a conservative prevalence of neurologic impairments of 6.1% in the community.^17^ We assumed that neurologic impairments would have a substantial overlap or comorbidity with developmental conditions such as ADHD and ASD. A simple random sampling method was used to select eligible children.

### Study Design and Procedures

This study used a 2-stage design: stage I involved screening for NDDs in the community. Children with a positive screen result in stage I were invited for further clinical, neuropsychological, and mental health assessment in stage II. In addition, every fifth child with a negative screen result in stage I was invited for clinical evaluation and neuropsychological and mental health assessments to assess the validity of screening tools and to obtain information for analysis of risk factors. The Neurodevelopmental Screening Tool (NDST) was administered to the parents or close caregivers of each eligible child in stage I^18,19^ from March 16, 2015, to September 14, 2016. The NDST includes questions for 7 domains of NDDs (ADHD, ASD, epilepsy, intellectual disability, and impairments in motor, hearing, and visual function) (eTable 1 in Supplement 1),^19^ but children with a positive screening result for any questions in 1 domain were again assessed for all disorders in stage II. The NDST detects any NDD with a sensitivity of 87.8% (95% CI, 87.3%-88.5%) and a specificity of 82.8% (95% CI, 82.1%-83.5%).^19^ Validity measures for individual domains of NDDs are shown in eTable 2 in Supplement 1.

In stage II, trained clinicians and neuropsychological assessors obtained a clinical history and performed a clinical examination. Cognition was measured with the Ravens Colored Progressive Matrix Test and Kilifi Naming Test,^15^ ADHD with the Kiddie Schedule for Affective Disorders and Schizophrenia for School-Age Children–Present and Lifetime (K-SADS-PL),^16^ and ASD with the Autism Diagnostic Observation Schedule (ADOS) or Developmental Diagnostic Dimensional Interview (3Di)^20^ following *Diagnostic and Statistical Manual of Mental Disorders* (Fifth Edition) (*DSM-5)* criteria. All the tools, including the NDST, were translated into the local languages, Kiswahili or Kigiriama, through a standardized forward-and-back translation process with cultural and conceptual adaptation and evaluation of psychometric properties. Focused group discussions and in-depth interviews were conducted with adults in the community to elicit phrases and idioms to be used in the translated version. The assessment tools were piloted to test their appropriateness in assessing neurodevelopment and were adapted accordingly before use in the epidemiologic survey. The tools were administered by experienced and trained neuropsychological assessors supervised by a developmental psychologist (A.A.), a child and adolescent psychiatrist (J.P.O.), a neurologist (C.R.J.C.N.), and an epidemiologist (S.M.K.).

A risk factor questionnaire was given to the parents of every child assessed at stage II (ie, children with a positive screen result in stage I and every fifth child with a negative screen result in stage I). The risk factor questionnaire had items on socioeconomic status, medical history, and child habits that may accelerate the onset of an NDD, consistent with the double- or triple-hit hypothesis.^10^ The questionnaire was designed for use in neurodevelopmental studies following a thorough review for possible risk factors for neurodevelopment. Electroencephalography was performed on children with a history of seizures according to the International 10-20 System, following the same preparation, duration, and hyperactivation protocols previously described.^21^

### Diagnosis and Definitions

ADHD was diagnosed using the ADHD module of the K-SADS-PL, which has excellent psychometric properties^16^ and requires parent-child participation, although we relied mostly on the parent’s or caretaker’s accounts of the child’s behavior. In the K-SADS-PL screening interview, a response was rated as either absent (coded as 1), at a subthreshold level (coded as 2), or at a threshold level (coded as 3). In the diagnostic K-SADS-PL supplement, ratings were given on a scale of 0 (no information), 1 (no ADHD symptoms), or 2 (presence of ADHD symptoms). ASD was diagnosed using the 3Di scoring algorithm and by clinical judgement using the *DSM-5* criteria and ADOS videos.^20,22^ Cognitive ability (as a marker of intellectual disability) was considered impaired if the child had a standardized *Z* score of less than 2 on either the Ravens Test or the Kilifi Naming Test. Epilepsy was defined as a history of 2 or more unprovoked seizures according to recommendations by the International League Against Epilepsy,^23^ and febrile seizures were identified and excluded as previously reported.^24^ Motor deficits were considered present if a child was unable to hold objects, sit, stand upright, walk, or put on clothes at an appropriate age. Poor scholastic achievement was defined as parental reports of a child’s nonattendance at school or poor school participation (eg, absenteeism) and poor academic performance (eg, being in a lower class grade or year than same-age peers). In this study, epilepsy and impairments in motor function, vision, and hearing function were considered neurologic conditions, while ASD and ADHD were considered developmental conditions. Prevalence was operationally defined as the proportion of children aged 6 to 9 years with any of the 7 domains of NDDs (ADHD, ASD, cognitive impairment, epilepsy, and impairments in motor, hearing, and visual functions). History of epilepsy was assessed as of the day the epidemiologic survey was conducted (eTable 3 in Supplement 1).

### Statistical Analysis

Data were entered using the MySQL open-source database, version 5 (Oracle Corporation), and analyzed using Stata, version 17.0 (StataCorp LLC). Prevalence was computed by dividing observed cases by the total population screened in stage I, specifying binomial exact 95% CIs. Standardized *Z* scores for assessment tests were generated by subtracting mean scores of a representative sample from the individual scores and dividing by the SD of the representative sample. The representative sample for generating means and SDs was created by restricting the background impairments as screened by the NDST to the expected prevalence in the general population. The prevalence accounted for sensitivity and specificity of the screening tools at stage I and for attrition (proportion of those who failed to turn up in stage II for further clinical evaluation after up to 3 invitations) between stage I and II.

Sex-specific sensitivities for prevalence estimates were stratified by sex. Log binomial regression was used to compute prevalence ratios for prevalence estimates by sex and age group. Generalized linear models specified for a log link and binomial family were used to compute the risk ratios (RRs) of the factors associated with NDDs; the analysis was repeated for neurologic disorders only and developmental conditions separately. We classified risk factors collected in stage II into 3 major categories (pregnancy and birth information, medical history information, and socioeconomic information) and then constructed an intermediary model for each individual risk factor that was adjusted both for child variables (age, sex, and schooling) and for maternal variables (marital status, ethnicity, and religion). For each of the 3 categories of risk factors, all variables reaching a 2-sided *P* value of less than .25 were entered into a multivariable model for that category to examine independent factors associated with NDDs. A test for linear trend was performed for risk factors categorized into 3 or more levels. We further examined the medical comorbidities of NDDs using generalized linear models as specified previously, adjusted only for age and sex of the child. A sensitivity analysis was done to investigate if independent factors associated with NDDs were shared between developmental conditions and neurologic conditions or were unique for each condition. Discrete variables were compared using Pearson χ^2^ or Fisher exact tests where observations in a cell were sparse (fewer than 5). Statistical significance was set at 2-sided *P* < .05.

## Results

### General Description

A total of 11 223 children were screened in stage I, of whom 5577 (49.7%) were females, 5646 (50.3%) were males, and 2361 (21.0%) had a positive NDST screen result in stage I. Mean (SD) participant age was 7.6 (0.96) years. A total of 2212 children (19.7%) were seen in stage II of the study, comparing 1653 (70.0%) of the 2361 who had a positive screen result in stage I and 559 (6.3%) of the 8862 with a negative screen result in stage I, selected randomly (every fifth child) (Figure 1). There were no statistically significant differences between children with a positive screen result who were assessed (n = 1653 [70.0%]) and those with a positive result who were not assessed (n = 708 [30.0%]) in terms of age (median, 7 years [range, 6-9 years] vs 8 years [range, 6-9 years], respectively; *Z* = 1.31; *P* = .19) and sex (assessed: females, 771 [46.6%]; males, 882 [53.4%]; not assessed: females, 313 [44.2%]; males, 395 [55.8%]; χ^2^ = 1.18; *P* = .28).

### Absolute Numbers and Overlap of Neurodevelopmental Disorders

There were 522 children with any of the 7 NDDs, with the highest proportions having ADHD (285 [54.5%]) and cognitive impairment (148 [28.3%]), while few children had visual impairments (14 [2.7%]). A significantly higher proportion of males than females had NDDs (285 [5.0%] vs 237 [4.2%]; χ^2^ = 4.03; *P* = .045), which was largely attributable to the preponderance of males with ADHD (167 [3.0%], compared with 118 females [2.1%]; χ^2^ = 5.18; *P* = .02) (eTable 4 in Supplement 1). The proportion with ASD was similar between females (50 [0.9%]) and males (46 [0.8%]) (χ^2^ = 0.22; *P* = .64), and so was the distribution for cognitive impairment in females with ASD (11 [22.0%]) and males with ASD (17 [37.0%]) (χ^2^ = 2.59; *P* = .11). Occurrence of comorbidity (>1 NDD) was found in 118 of the children with any NDDs (22.6%; 95% CI, 19.2%-26.5%), while in the remainder, an NDD occurred as a single condition. The number of NDDs that overlapped ranged from 2 to 5, with the overlap of 2 conditions (92 children [17.3%; 95% CI, 14.1%-20.8%]) and 3 conditions (16 children [3.1%; 95% CI, 1.8%-4.9%]) being the most common and 5 conditions the least (1 child [0.2%; 95% CI, 0.0%-1.1%]) (eFigure in Supplement 1).

### Prevalence of Neurodevelopmental Disorders

The overall prevalence for any NDD was 9.1% (90.8 [95% CI, 83.4-98.6] cases per 1000 children), after adjusting for attrition between stages I and II and sensitivity and specificity of the NDST in stage I. The adjusted prevalence was 44.8 (95% CI, 39.5-50.6) cases per 1000 children for any neurologic condition and 59.7 (95% CI, 53.9-66.4) cases per 1000 children for ASD or ADHD. The individual NDD domain with the highest adjusted prevalence was ADHD (50.8 [95% CI, 45.2-57.1] cases per 1000 children), followed by cognitive impairment (27.1 [95% CI, 22.9-31.9] cases per 1000 children) and ASD (15.6 [95% CI, 12.6-19.1] cases per 1000 children), with visual impairment having the lowest prevalence (1.8 [95% CI, 0.9-3.4] cases per 1000 children) (Table 1).

**Table 1.** Prevalence of Neurodevelopmental Disorders Per 1000 Children

Neurodevelopmental disorder	Children, raw No.	Crude prevalence (95% CI)	Adjusted prevalence (95% CI)^a^
ADHD	285	25.4 (22.6-28.5)	50.8 (45.2-57.1)
ASD	96	8.5 (7.0-10.4)	15.6 (12.6-19.1)
Cognitive impairment	148	13.2 (11.2-15.5)	27.1 (22.9-31.9)
Epilepsy	98	8.7 (7.1-10.6)	17.6 (14.2-21.4)
Motor impairment	18	1.6 (1.0-2.6)	2.5 (1.4-4.3)
Hearing impairment	18	1.6 (1.0-2.6)	2.5 (1.4-4.3)
Visual impairment	14	1.2 (0.7-2.1)	1.8 (0.9-3.4)
Any neurologic impairment	251	22.4 (19.8-25.3)	44.8 (39.5-50.6)
Any ASD or ADHD	344	30.6 (27.6-34.0)	59.7 (53.9-66.4)
Any neurodevelopmental disorder	522	46.5 (42.7-50.6)	90.8 (83.4-98.6)

Abbreviations: ADHD, attention-deficit/hyperactivity disorder; ASD, autism spectrum disorder.

^a^ Adjusted for attrition between stages I and II of the study and for the sensitivity and specificity of the screening questionnaire.

### Comorbidity of Developmental Conditions With Neurologic Conditions

After adjustment, ADHD was significantly associated with ASD (adjusted RR [ARR], 3.25; 95% CI, 2.46-4.29). The risk of ASD being comorbid with any neurologic condition was significant (ARR, 4.70; 95% CI, 3.17-6.96) and was greatest for cognitive impairment (ARR, 5.76; 95% CI, 3.83-8.66) and epilepsy (ARR, 4.00; 95% CI, 2.41-6.64) (Table 2). The risk of ADHD being comorbid with other neurologic impairments was significant (ARR, 1.77; 95% CI, 1.36-2.32), and when considering individual domains, the risk of comorbidity was greatest for epilepsy (ARR, 2.44; 95% CI, 1.76-3.37) and cognitive impairment (ARR, 1.76; 95% CI, 1.27-2.44). Having a developmental condition (ASD or ADHD) was associated with having any neurologic condition (ARR, 2.10; 95% CI, 1.68-2.62), and the risk of comorbidity between ASD or ADHD and individual neurologic domains was greatest for epilepsy (ARR, 2.45; 95% CI, 1.84-3.25) and cognitive impairment (ARR, 2.20; 95% CI, 1.70-2.85). Cognitive impairment was also associated with reduced attendance at school (ARR, 0.81; 95% CI, 0.74-0.88). Having a developmental condition was not associated with impairments in motor, hearing, and vision function (Table 2).

**Table 2.** Overlap of Specific Neurologic Conditions With Specific Developmental Conditions Among Children Assessed for Neurodevelopmental Disorders in Stage II of the Study

Neurologic condition	ASD			ADHD			Developmental condition^a^		
	No, No. (%) (n = 2116)	Yes, No. (%) (n = 96)	ARR (95% CI)^b^	No, No. (%) (n = 1927)	Yes, No. (%) (n = 285)	ARR (95% CI)^b^	No, No. (%) (n = 1868)	Yes, No. (%) (n = 344)	ARR (95% CI)^b^
Cognitive impairment	120 (5.7)	28 (29.2)	5.76 (3.83-8.66)	116 (6.0)	32 (11.2)	1.76 (1.27-2.44)	101 (5.4)	47 (13.7)	2.20 (1.70-2.85)
Epilepsy	83 (3.9)	15 (15.6)	4.00 (2.41-6.64)	66 (3.6)	29 (10.2)	2.44 (1.76-3.37)	63 (3.4)	35 (10.2)	2.45 (1.84-3.25)
Motor impairment	16 (0.8)	2 (2.1)	2.48 (0.65-1.23)	15 (0.8)	3 (1.1)	1.39 (0.49-3.93)	13 (0.8)	5 (1.5)	1.87 (0.87-3.99)
Hearing impairment	18 (0.9)	0	0.96 (0.95-0.97)	17 (0.9)	1 (0.4)	0.44 (0.06-2.92)	17 (0.9)	1 (0.3)	0.36 (0.05-2.39)
Visual impairment	13 (0.6)	1 (1.2)	1.67 (0.25-11.19)	12 (0.6)	2 (0.7)	1.04 (0.29-3.81)	11 (0.6)	3 (0.9)	1.34 (0.49-3.69)
Any neurologic impairment	215 (10.2)	36 (37.5)	4.70 (3.17-6.96)	198 (10.3)	53 (18.6)	1.77 (1.36-2.32)	178 (9.5)	73 (21.4)	2.10 (1.68-2.62)

Abbreviations: ADHD, attention-deficit/hyperactivity disorder; ARR, adjusted risk ratio; ASD, autism spectrum disorder.

^a^ Having a developmental condition (ASD or ADHD) was entered into the models as a response variable and neurologic conditions were entered as explanatory variables.

^b^ RRs were adjusted a priori for age and sex.

### Factors Associated With Having Any Neurodevelopmental Disorder

Two pregnancy and birth factors, adverse perinatal events and substance use or abuse, were associated with the risk of any NDD in the multivariable models, with adverse perinatal events associated with the greatest risk (ARR, 1.64; 95% CI, 1.29-2.09) and substance use or abuse associated with a reduced risk (ARR, 0.68; 95% CI, 0.46-0.99) (eTable 5 in Supplement 1). There were 6 medical history factors associated with having any NDD in multivariable models; the leading factors were history of previous hospitalization (ARR, 1.40; 95% CI, 1.18-1.66) and snoring more than 3 times a week (ARR, 1.50; 95% CI, 1.27-1.76), but febrile infections (ARR, 1.28; 95% CI, 1.05-1.56) and eating soil (ARR, 1.45; 95% CI, 1.13-1.85), along with head injury and use of a bed net, were also associated with NDDs. Among the socioeconomic factors, being landless (ARR, 1.32; 95% CI, 1.12-1.56) and having dead siblings (ARR, 1.11; 95% CI, 1.03-1.19) were associated with having any NDDs.

### Differential Factors Associated With Developmental Conditions and With Neurologic Conditions

**Figure 2.**
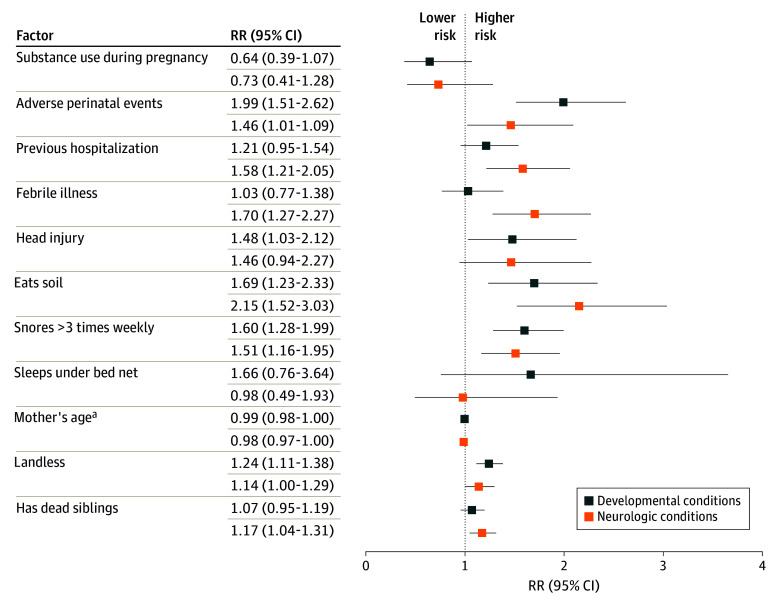
Multivariable Risk Ratios (RRs) of Factors Associated With Developmental Conditions and With Neurologic Conditions A sensitivity analysis was done for factors reaching multivariable significance thresholds for all neurodevelopmental disorders. ^a^Reference category for mother’s age was 19 years or older.

Stratified analysis of risk factors that met the significance threshold (*P* < .25) for the multivariable model of NDD risk by developmental and neurologic conditions showed shared factors associated with these conditions, namely eating soil (RR, 1.69 [95% CI, 1.23-2.33] for developmental and 2.15 [95% CI, 1.52-3.03] for neurologic conditions), along with adverse perinatal events (RR, 1.99 [95% CI, 1.51-2.62] for developmental and 1.46 [95% CI, 1.01-1.09] for neurologic conditions) and being landless (RR, 1.24 [95% CI, 1.11-1.38] for developmental and 1.14 [95% CI, 1.00-1.29] for neurologic conditions). Factors associated with neurologic conditions alone included febrile illnesses (RR, 1.70; 95% CI, 1.27-2.27), along with previous hospitalization (RR, 1.58; 95% CI, 1.21-2.05) and having dead siblings (RR, 1.17; 95% CI, 1.04-1.31). Head injury (RR, 1.48; 95% CI, 1.03-2.12) was the only factor significantly associated with risk of developmental conditions alone (Figure 2 and eTable 6 in Supplement 1).

### Medical Comorbidities of Neurodevelopmental Disorders

Medical comorbidities that were more frequent in those with any NDD included motor neuron problems (measured from reflexes [ie, involuntary body responses to a stimulus, used to locate motor neuron lesions]) (ARR, 2.79; 95% CI, 2.18-3.56), skin bruises (ARR, 1.46; 95% CI, 1.04-2.06), and malnutrition (as measured from stunting and wasting, defined as either height-for-age or weight-for-age *Z* score of 2 or lower) (ARR, 1.80; 95% CI, 1.37-2.39). Reduced sensation to touch was less frequent in those with NDDs (ARR, 0.57; 95% CI, 0.35-0.91) (Table 3). School nonattendance was associated with motor neuron problems (ARR, 6.05; 95% CI, 3.27-11.21), skin bruises (ARR, 3.45; 95% CI, 1.73-6.89), and malnutrition (ARR, 3.62; 95% CI, 1.79-7.34) but not with sensation problems (ARR, 1.05; 95% CI, 0.43-2.55). No association was observed between school nonattendance and socioeconomic variables, specifically being landless (ARR, 1.09; 95% CI, 0.87-1.35).

**Table 3.** Associations of Medical Comorbidities With Neurodevelopmental Disorders as Diagnosed in Stage II of the Study

Medical comorbidity^a^	Children, No./total No. (%)		ARR (95% CI)^b^	*P* value
	Without neurodevelopmental disorder (n = 1690)	With neurodevelopmental disorder (n = 522)		
Respiratory problems	11/1643 (0.7)	5/504 (1.0)	1.33 (0.64-2.75)	.45
Self-reported HIV infection	6/1607 (0.4)	4/497 (0.8)	1.73 (0.81-3.68)	.16
Lymphadenopathy	15/1629 (0.9)	7/502 (1.4)	1.35 (0.73-2.50)	.34
Sickle cell disease	7/1605 (0.4)	5/495 (1.0)	1.76 (0.89-3.45)	.10
Cardiovascular problems	7/1641 (0.4)	3/502 (0.6)	1.24 (0.48-3.17)	.66
Burns	109/1633 (6.7)	31/511 (6.1)	0.92 (0.67-1.26)	.60
Skin bruises	45/1636 (2.8)	23/505 (4.6)	1.46 (1.04-2.06)	.03
Motor neuron problems	15/1644 (0.9)	26/506 (5.1)	2.79 (2.18-3.56)	<.001
Sensation problems	82/852 (9.6)	15/301 (5.0)	0.57 (0.35-0.91)	.02
Malnutrition^c^	60/398 (15.1)	44/148 (29.7)	1.80 (1.37-2.39)	<.001
Microcephaly^d^	150/395 (38.0)	62/149 (41.6)	1.16 (0.88-1.54)	.30
Otitis media infections	34/1638 (2.1)	15/501 (3.0)	1.32 (0.86-2.03)	.20

Abbreviation: ARR, adjusted risk ratio.

^a^ Medical comorbidities were collected during stage II of the study.

^b^ Adjusted for age and sex.

^c^ Defined as either height-for-age or weight-for-age *Z* score of 2 or lower.

^d^ Defined as head-circumference-for-age *Z* score of 2 or lower.

## Discussion

To our knowledge, this is the first study to estimate the prevalence of all NDDs in a region of Africa, and it suggests that the epidemiologic burden is enormous. The burden of mental conditions was greater than that of neurologic conditions, although these conditions often occurred together, with 1 child having 5 disorders. The findings suggest there are several factors that may prime the occurrence of NDDs, even in children with underlying genetic susceptibility, from across the pregnancy and birth, medical history, and socioeconomic categories. Several conditions were comorbid with NDDs, top among them being scholastic problems, reflex problems, and poor nutritional status.

The burden of NDDs from this study (90.8 cases per 1000 children) was higher than in studies that looked at neurologic impairments only (61 cases per 1000 children).^13,25^ Adding developmental conditions in our study increased estimates from surveys of neurologic conditions done in 2001^13,25^ by only 30 cases per 1000 children, perhaps because of the substantial overlap between the conditions. Although neonatal conditions have increased, intracranial infections have reduced, which may lower estimates for neurologic disorders such as epilepsy.^25^ Meta-analytic estimates from individual NDDs weight the burden downward because epilepsy is more studied than ASD and ADHD in LMICs.^6^ Our NDD prevalence estimates are comparable to estimates from India (9.2%-13.8%).^7^ That Asian study, however, was based on a smaller sample drawn from across several heterogeneous districts (both rural and urban) and used a denominator population from a national census, which is less reliable than from demographic surveillance systems.

The prevalence of NDDs in this study was highest for ADHD (50.8 cases per 1000 children), similar to estimates of 5.2% and 4.8% from Germany^26,27^ but slightly lower than the 7.3% reported in Ethiopia^28^; differences in study designs and assessment tools used may explain these variations. ASD estimates were similar to those in the Indian study,^7^ but in our study, the proportions of females and males were similar. Distribution of cognitive impairment was inversely proportional to prevalence of ASD by sex in older children (aged 8 years) from the US,^29^ a relationship that may explain reduced cognitive impairment in males with ASD in our study, in whom cognitive impairment appeared to be more frequent than in females. Sex-ratio differences in ASD are also associated with sex-linked mutational burden and copy number variants.^30^ As in previous studies,^14,31^ intellectual disability and epilepsy were the most reported neurologic conditions in our study, compared with vision and hearing, which are associated with significant disability^32,33^ but are likely to be underreported during screening.

There was strong evidence that NDDs were comorbid with each other, with overlap noted in 22.6% of children. ASD and ADHD were associated with neurologic conditions, as in a study from a high-income country.^34^ Studies in LMICs reported this neurobehavioral comorbidity in older children with epilepsy^24^ and in preschool children with acute seizures^35^ but not across all NDDs. Preschool mental health problems can persist into school age. Hearing impairments in this study were infrequently reported among children with NDDs, in some of whom auditory sensitivity is common.^36^ As expected, children with cognitive impairments were also likely to miss school. These findings encourage assessment of different domains of NDDs in a child and development of a comprehensive management plan.

There were several factors that may prime the occurrence of NDDs in this region and that can be targeted for interventions. Medical history of conditions that can directly cause damage to the brain (eg, acute febrile illnesses, adverse perinatal events, and head injury) were factors associated with NDDs. Febrile illnesses with neurologic involvement during hospital admission^37^ can result in neurobehavioral problems that persist for years following discharge.^38^ In this study, febrile illnesses were more specific to neurologic conditions such as epilepsy, possibly related to damage of cortical structures, while adverse perinatal events were salient for mental health conditions, likely related to damage of deep brain structures (eg, the thalamus).

Snoring, eating soil, and using a bed net are emerging as factors associated with NDDs, including in preschool children from the Kenya area.^9^ Eating soil with heavy metals or intestinal worms may result in brain dysfunction,^39^ although NDDs may cause children to eat soil. Similarly, upper airway obstruction, which may manifest as snoring, has been implicated in mental health problems in children.^9^ Risk for NDDs in children using bed nets may be related to earlier exposure to malaria, although concerns about the neurotoxic effects of permethrin should be dispelled. Contrary to evidence,^40^ use of drugs in pregnancy was associated with reduced risk of NDDs and may be a proxy for mothers who visited and used biomedical facilities and medicine, which can be beneficial during pregnancy.

Socioeconomic factors may be a marker for social disadvantage or clustering of family or environmental adversity that can lead to NDDs. These factors together may lead to NDDs through social causation (eg, limiting access to health) or social selection (less productivity and poverty related to having the disorder). It is unclear if the associations between young maternal age and NDDs such as ASD are biologically relevant or reflect confounding by socioeconomic disadvantage of young mothers.^41^

Children with NDDs experience other medical problems. In this study, skin bruises were common in children with NDDs, similar to typical skin injuries seen in children with ASD.^42^ Reflex and sensory problems or symptoms in the children with NDDs perhaps represent a previously recognized dysfunctional motor neuron system or hyporesponsiveness to stimuli.^43,44^ The association of malnutrition with NDDs should be clarified in future studies, since nutritional status may affect neurodevelopment, which also influences access to food and poor feeding and appetite.^45^ Addressing these medical comorbidities may improve school attendance in some children.

Findings from this rural area of Kenya should be compared with those from urban areas. The rate of urbanization in Africa is the highest in the world. This results in unemployment, overcrowding, and exposure to infectious and environmental hazards,^46^ likely influencing the burden of NDDs in urban settings in Africa.

### Strengths and Limitations

This study has strengths, including its large size and use of validated and standardized tests to diagnose NDDs and to identify risk factors. Estimates are reliable because they were adjusted for attrition and sensitivity or specificity of the screening tools, although underestimation is possible due to stigma and underappreciation of symptoms of NDDs in this area of Kenya.

The study also has limitations. There are NDDs that were not assessed in this study (eg, chronic tic disorders, Tourette syndrome), and more children with negative screening results could not be assessed in stage II because of logistic constraints. There were no data to test concordance between responses from parents and those from other caregivers. This cross-sectional study was also unable to establish causality of NDDs with regard to risk factors or comorbid conditions. Residual confounding by unmeasured factors cannot be ruled out, and use of self-reports may be subject to recall bias.

## Conclusions

The burden of NDDs among children in this cross-sectional study in Kenya was as high as in other low-income settings,^7^ and the findings suggest it may be addressed through targeted control of modifiable risk factors that occur antenatally and postnatally. There is a need to screen for all NDDs in children during developmental monitoring and screening. A comprehensive management plan for NDDs should be developed that incorporates other medical problems. Future studies are needed to understand the relationship between comorbid neurodevelopmental disorders and their associated factors at the spatial level to improve the stakeholders’ understanding of conditions such as ASD and to support parents of affected children.

## Notes

Supplement 1. **eTable 1.** Neurodevelopmental Screening Tool (NDST) Questions for Domains of Neurodevelopmental Disorders and Their Possible Overlap **eTable 2.** Validity of All NDST Questions in Detecting Neurodevelopmental Disorders **eTable 3.** Definitions of Neurodevelopmental Disorders **eTable 4.** Children With Neurodevelopmental Disorders According to Age and Sex **eTable 5.** Risk Factors for All Neurodevelopmental Disorders (NDDs) Diagnosed in Stage II **eTable 6.** Multivariable Risk Ratios (RRs) of Factors Associated With Developmental Conditions and With Neurologic Conditions **eFigure.** Overlap Distribution by Number of Neurodevelopmental Disorders
eReference

Supplement 2.
Data Sharing Statement

## References

[1] Murray CJ, Vos T, Lozano R, . Disability-adjusted life years (DALYs) for 291 diseases and injuries in 21 regions, 1990-2010: a systematic analysis for the Global Burden of Disease Study 2010. Lancet. 2012;380(9859):2197-2223. doi:10.1016/S0140-6736(12)61689-4 23245608

[2] Thickbroom GW, Mastaglia FL. Plasticity in neurological disorders and challenges for noninvasive brain stimulation (NBS). J Neuroeng Rehabil. 2009;6:4. doi:10.1186/1743-0003-6-4 19222843 PMC2649147

[3] Collins PY, Patel V, Joestl SS, ; Scientific Advisory Board and the Executive Committee of the Grand Challenges on Global Mental Health. Grand challenges in global mental health. Nature. 2011;475(7354):27-30. doi:10.1038/475027a 21734685 PMC3173804

[4] Newton CR. Neurodevelopmental disorders in low- and middle-income countries. Dev Med Child Neurol. 2012;54(12):1072. doi:10.1111/j.1469-8749.2012.04384.x 22803812

[5] Garner RE, Arim RG, Kohen DE, . Parenting children with neurodevelopmental disorders and/or behaviour problems. Child Care Health Dev. 2013;39(3):412-421. doi:10.1111/j.1365-2214.2011.01347.x 22066574

[6] Bitta M, Kariuki SM, Abubakar A, Newton CRJC. Burden of neurodevelopmental disorders in low and middle-income countries: a systematic review and meta-analysis. Wellcome Open Res. 2017;2:121. doi:10.12688/wellcomeopenres.13540.1 29881784 PMC5964629

[7] Arora NK, Nair MKC, Gulati S, . Neurodevelopmental disorders in children aged 2-9 years: population-based burden estimates across five regions in India. PLoS Med. 2018;15(7):e1002615. doi:10.1371/journal.pmed.1002615 30040859 PMC6057634

[8] Olusanya BO, Davis AC, Wertlieb D, ; Global Research on Developmental Disabilities Collaborators. Developmental disabilities among children younger than 5 years in 195 countries and territories, 1990-2016: a systematic analysis for the Global Burden of Disease Study 2016. Lancet Glob Health. 2018;6(10):e1100-e1121. doi:10.1016/S2214-109X(18)30309-7 30172774 PMC6139259

[9] Kariuki SM, Abubakar A, Kombe M, . Burden, risk factors, and comorbidities of behavioural and emotional problems in Kenyan children: a population-based study. Lancet Psychiatry. 2017;4(2):136-145. doi:10.1016/S2215-0366(16)30403-5 28137381 PMC5285446

[10] Guma E, Cupo L, Ma W, . Investigating the “two-hit hypothesis”: effects of prenatal maternal immune activation and adolescent cannabis use on neurodevelopment in mice. Prog Neuropsychopharmacol Biol Psychiatry. 2023;120:110642. doi:10.1016/j.pnpbp.2022.110642 36150422

[11] UNICEF. Committing to child survival: a promise renewed. September 8, 2015. Accessed November 4, 2025. https://data.unicef.org/resources/committing-to-child-survival-a-promise-renewed-2015/

[12] Kariuki SM, Abubakar A, Kombe M, . Prevalence, risk factors and behavioural and emotional comorbidity of acute seizures in young Kenyan children: a population-based study. BMC Med. 2018;16(1):35. doi:10.1186/s12916-018-1021-y 29510713 PMC5840716

[13] Mung’ala-Odera V, Meehan R, Njuguna P, Mturi N, Alcock KJ, Newton CRJC. Prevalence and risk factors of neurological disability and impairment in children living in rural Kenya. Int J Epidemiol. 2006;35(3):683-688. doi:10.1093/ije/dyl023 16492712

[14] Ngugi AK, Bottomley C, Kleinschmidt I, ; SEEDS group. Prevalence of active convulsive epilepsy in sub-Saharan Africa and associated risk factors: cross-sectional and case-control studies. Lancet Neurol. 2013;12(3):253-263. doi:10.1016/S1474-4422(13)70003-6 23375964 PMC3581814

[15] Kitsao-Wekulo PK, Holding PA, Taylor HG, Abubakar A, Connolly K. Neuropsychological testing in a rural African school-age population: evaluating contributions to variability in test performance. Assessment. 2013;20(6):776-784. doi:10.1177/1073191112457408 22936783

[16] Kariuki SM, Newton CRJC, Abubakar A, Bitta MA, Odhiambo R, Phillips Owen J. Evaluation of psychometric properties and factorial structure of ADHD module of K-SADS-PL in children from rural Kenya. J Atten Disord. 2020;24(14):2064-2071. doi:10.1177/1087054717753064 29392964 PMC7549293

[17] Mung’ala-Odera V, White S, Meehan R, . Prevalence, incidence and risk factors of epilepsy in older children in rural Kenya. Seizure. 2008;17(5):396-404. doi:10.1016/j.seizure.2007.11.028 18249012 PMC3428880

[18] Khan NZ, Muslima H, Shilpi AB, . Validation of a home-based neurodevelopmental screening tool for under 2-year-old children in Bangladesh. Child Care Health Dev. 2013;39(5):643-650. doi:10.1111/j.1365-2214.2012.01393.x 22676392

[19] Bitta MA, Kipkemoi P, Kariuki SM, . Validity and reliability of the Neurodevelopmental Screening Tool (NDST) in screening for neurodevelopmental disorders in children living in rural Kenyan coast. Wellcome Open Res. 2021;6:137. doi:10.12688/wellcomeopenres.16765.1 34676305 PMC8503789

[20] Kipkemoi P, Kariuki SM, Gona J, . Utility of the 3Di short version in the identification and diagnosis of autism in children at the Kenyan coast. Front Psychiatry. 2024;15:1234929. doi:10.3389/fpsyt.2024.1234929 38487576 PMC10937349

[21] Kariuki SM, White S, Chengo E, . Electroencephalographic features of convulsive epilepsy in Africa: a multicentre study of prevalence, pattern and associated factors. Clin Neurophysiol. 2016;127(2):1099-1107. doi:10.1016/j.clinph.2015.07.033 26337840 PMC4725253

[22] Akshoomoff N. Use of the Mullen Scales of Early Learning for the assessment of young children with autism spectrum disorders. Child Neuropsychol. 2006;12(4-5):269-277. doi:10.1080/09297040500473714 16911972 PMC1550495

[23] Thurman DJ, Beghi E, Begley CE, ; ILAE Commission on Epidemiology. Standards for epidemiologic studies and surveillance of epilepsy. Epilepsia. 2011;52(suppl 7):2-26. doi:10.1111/j.1528-1167.2011.03121.x 21899536

[24] Kind CJ, Newton CRJC, Kariuki SM; Neurodevelopment Disorders Study Group. Prevalence, risk factors, and neurobehavioral comorbidities of epilepsy in Kenyan children. Epilepsia Open. 2017;2(4):388-399. doi:10.1002/epi4.12069 29588970 PMC5862110

[25] Abuga JA, Kariuki SM, Abubakar A, . Neurological impairment and disability in children in rural Kenya. Dev Med Child Neurol. 2022;64(3):347-356. doi:10.1111/dmcn.15059 34536290 PMC9292953

[26] Huss M, Hölling H, Kurth BM, Schlack R. How often are German children and adolescents diagnosed with ADHD? prevalence based on the judgment of health care professionals: results of the German Health and Examination Survey (KiGGS). Eur Child Adolesc Psychiatry. 2008;17(suppl 1):52-58. doi:10.1007/s00787-008-1006-z 19132304

[27] Polanczyk G, de Lima MS, Horta BL, Biederman J, Rohde LA. The worldwide prevalence of ADHD: a systematic review and metaregression analysis. Am J Psychiatry. 2007;164(6):942-948. doi:10.1176/ajp.2007.164.6.942 17541055

[28] Lola HM, Belete H, Gebeyehu A, Zerihun A, Yimer S, Leta K. Attention deficit hyperactivity disorder (ADHD) among children aged 6 to 17 years old living in Girja District, rural Ethiopia. Behav Neurol. 2019;2019:1753580. doi:10.1155/2019/1753580 31110594 PMC6487158

[29] Developmental Disabilities Monitoring Network Surveillance Year 2010 Principal Investigators; Centers for Disease Control and Prevention (CDC). Prevalence of autism spectrum disorder among children aged 8 years—Autism and Developmental Disabilities Monitoring Network, 11 sites, United States, 2010. MMWR Surveill Summ. 2014;63(2):1-21. 24670961

[30] Polyak A, Rosenfeld JA, Girirajan S. An assessment of sex bias in neurodevelopmental disorders. Genome Med. 2015;7(1):94. doi:10.1186/s13073-015-0216-5 26307204 PMC4549901

[31] Mung’Ala-Odera V, Snow RW, Newton CR. The burden of the neurocognitive impairment associated with *Plasmodium falciparum* malaria in sub-Saharan Africa. Am J Trop Med Hyg. 2004;71(2)(suppl):64-70. doi:10.4269/ajtmh.2004.71.64 15331820

[32] Wilson BS, Tucci DL, Merson MH, O’Donoghue GM. Global hearing health care: new findings and perspectives. Lancet. 2017;390(10111):2503-2515. doi:10.1016/S0140-6736(17)31073-5 28705460

[33] Flaxman SR, Bourne RRA, Resnikoff S, ; Vision Loss Expert Group of the Global Burden of Disease Study. Global causes of blindness and distance vision impairment 1990-2020: a systematic review and meta-analysis. Lancet Glob Health. 2017;5(12):e1221-e1234. doi:10.1016/S2214-109X(17)30393-5 29032195

[34] Reilly C, Atkinson P, Das KB, . Neurobehavioral comorbidities in children with active epilepsy: a population-based study. Pediatrics. 2014;133(6):e1586-e1593. doi:10.1542/peds.2013-3787 24864167

[35] Kariuki SM, Abubakar A, Stein A, Marsh K, Newton CRJC. Prevalence, causes, and behavioral and emotional comorbidities of acute symptomatic seizures in Africa: a critical review. Epilepsia Open. 2017;2(1):8-19. doi:10.1002/epi4.12035 29750209 PMC5939456

[36] Lucker JR. Auditory hypersensitivity in children with autism spectrum disorders. Focus Autism Other Dev Disabl. 2013;28(3):184-191. doi:10.1177/1088357613475810

[37] Idro R, Ndiritu M, Ogutu B, . Burden, features, and outcome of neurological involvement in acute falciparum malaria in Kenyan children. JAMA. 2007;297(20):2232-2240. doi:10.1001/jama.297.20.2232 17519413 PMC2676709

[38] Carter JA, Mung’ala-Odera V, Neville BGR, . Persistent neurocognitive impairments associated with severe falciparum malaria in Kenyan children. J Neurol Neurosurg Psychiatry. 2005;76(4):476-481. doi:10.1136/jnnp.2004.043893 15774431 PMC1739592

[39] Lambein F, Haque R, Khan JK, Kebede N, Kuo YH. From soil to brain: zinc deficiency increases the neurotoxicity of *Lathyrus sativus* and may affect the susceptibility for the motorneurone disease neurolathyrism. Toxicon. 1994;32(4):461-466. doi:10.1016/0041-0101(94)90298-4 8053001

[40] Khemiri L, Kuja-Halkola R, Larsson H, . Parental substance use disorder and risk of intellectual disability in offspring in Sweden: a national register study. EClinicalMedicine. 2023;63:102170. doi:10.1016/j.eclinm.2023.102170 37680949 PMC10480548

[41] Sandin S, Schendel D, Magnusson P, . Autism risk associated with parental age and with increasing difference in age between the parents. Mol Psychiatry. 2016;21(5):693-700. doi:10.1038/mp.2015.70 26055426 PMC5414073

[42] Slingsby B, Yatchmink Y, Goldberg A. Typical skin injuries in children with autism spectrum disorder. Clin Pediatr (Phila). 2017;56(10):942-946. doi:10.1177/0009922817705187 28457143

[43] Baranek GT, David FJ, Poe MD, Stone WL, Watson LR. Sensory Experiences Questionnaire: discriminating sensory features in young children with autism, developmental delays, and typical development. J Child Psychol Psychiatry. 2006;47(6):591-601. doi:10.1111/j.1469-7610.2005.01546.x 16712636

[44] Williams ZJ, Schaaf R, Ausderau KK, . Examining the latent structure and correlates of sensory reactivity in autism: a multi-site integrative data analysis by the autism sensory research consortium. Mol Autism. 2023;14(1):31. doi:10.1186/s13229-023-00563-4 37635263 PMC10464466

[45] Kariuki SM, Matuja W, Akpalu A, ; SEEDS writing group. Clinical features, proximate causes, and consequences of active convulsive epilepsy in Africa. Epilepsia. 2014;55(1):76-85. doi:10.1111/epi.12392 24116877 PMC4074306

[46] Güneralp B, Lwasa S, Masundire H, Parnell S, Seto KC. Urbanization in Africa: challenges and opportunities for conservation. Environ Res Lett. 2017;13(1):015002. doi:10.1088/1748-9326/aa94fe

