# Supplementary material for: Burden of Neurodevelopmental Disorders in Kenyan Children
Source: JAMA Netw Open. 2025 Dec 12;8(12):e2548853. doi: 10.1001/jamanetworkopen.2025.48853 (PMC12701508; doi:10.1001/jamanetworkopen.2025.48853)
Supplement: Supplement 1. — eTable 1. Neurodevelopmental Screening Tool (NDST) Questions for Domains of Neurodevelopmental Disorders and Their Possible Overlap eTable 2. Validity of All NDST Questions in Detecting Neurodevelopmental Disorders eTable 3. Definitions of Neurodevelopmental Disorders eTable 4. Children With Neurodevelopmental Disorders According to Age and Sex eTable 5. Risk Factors for All Neurodevelopmental Disorders (NDDs) Diagnosed in Stage II eTable 6. Multivariable Risk Ratios (RRs) of Factors Associated With Developmental Conditions and With Neurologic Conditions eFigure. Overlap Distribution by Number of Neurodevelopmental Disorders eReference [file jamanetwopen-e2548853-s001.pdf]

Supplementary Online Content

Kariuki SM, Kipkemoi P, Kombe MZ, et al. Burden of neurodevelopmental disorders in Kenyan children. *JAMA Netw Open*. 2025;8(12):e2548853. doi:10.1001/jamanetworkopen.2025.48853

eTable 1. Neurodevelopmental Screening Tool (NDST) Questions for Domains of Neurodevelopmental Disorders and Their Possible Overlap
eTable 2. Validity of All NDST Questions in Detecting Neurodevelopmental Disorders
eTable 3. Definitions of Neurodevelopmental Disorders
eTable 4. Children With Neurodevelopmental Disorders According to Age and Sex
eTable 5. Risk Factors for All Neurodevelopmental Disorders (NDDs) Diagnosed in Stage II
eTable 6. Multivariable Risk Ratios (RRs) of Factors Associated With Developmental Conditions and With Neurologic Conditions
eFigure. Overlap Distribution by Number of Neurodevelopmental Disorders
eReference

This supplementary material has been provided by the authors to give readers additional information about their work.

© 2025 Kariuki SM et al. *JAMA Network Open*.

eTable 1. Neurodevelopmental Screening Tool (NDST) questions for domains of neurodevelopmental disorders and their possible overlap

Item	Questions	Domain
nds1	Does your child make common age appropriate gestures to greet familiar people	Autism, Intellectual Disability
nds2	Did /does your child ever seek your attention by pointing to thing	Autism, Intellectual Disability, Visual
nds3	Does your child look at your face and maintain eye contact when you are talking to him /her?	Autism, Intellectual Disability, Visual
nds4	Did/does your child ever engage in pretend plays or games which involve role play	Autism, Intellectual Disability, Motor, Visual
nds5	Like other children of his /her age, is your child able to do his /her activities of daily living by himself/ herself	Autism, Intellectual Disability, Motor, Visual
nds6	Does your child have difficulty in seeing clearly from a distance	Visual
nds7	Does your child have difficulty in seeing, either during day or night? (without spectacles)	Visual
nds8	Does your child have difficulty in seeing small objects?	Visual
nds9	Does your child bring objects very close to the eyes to see them?	Visual
nds10	Compared with other children of his /her age, did your child have any delay in walking?	Intellectual Disability, Motor
nds11	Does your child collide with objects while walking independently or has frequent falls while walking?	Motor, Visual
nds12	Does your child have difficulty in moving his /her limbs?	Motor*,
nds13	Does your child have difficulty in getting up from squatting position or has progressive worsening/ increasing difficulty in walking, running or climbing stairs	Motor,
nds14	Does your child have stiffness or floppiness and/or reduced strength in his /her arms or legs?	Motor
nds15	From birth till date, has your child ever had fits, or became rigid, or had sudden jerks or spasms of arms, legs or whole body?	Epilepsy
nds16	From birth till date, has your child ever lost consciousness?	Epilepsy
nds17	Did your child need change of school, or dropped out of school due to very poor performance?	Intellectual Disability, ADHD, LD
Nds18	Do the teachers complain about your child’s very poor performance in studies?	Intellectual Disability, ADHD, LD
nds19	Compared to his/ her classmates, does your child have significant difficulty in any of the subjects?	Intellectual Disability, ADHD, LD
nds20	Compared to his/ her classmates, does your child find it difficult to read or write or to do simple calculations?	Visual, LD
nds21	Can your child speak?	SLD
nds22	After attaining initial speech has your child now stopped speaking or has he/she stopped learning new words and sentences?	Autism, Intellectual Disability, SLD
nds23	Is your child's speech in any way different from other children of his/ her age?	Autism, Intellectual Disability, SLD
nds24	Can your child speak words or sentences which can also be understood by non-family members?	Intellectual Disability, SLD
nds25	Does your child often repeat the same word or phrase over and over again in the same manner?	Autism
nds26	Can your child name familiar objects (if less than 3 years old) or is able to describe an object /event or an action (if more than 3 years old)?	Autism, Intellectual Disability, SLD
nds27	Does your child have difficulty in hearing?	Hearing
nds28	Does your child pay attention when you address him/her by name?	Autism, Intellectual Disability, Hearing
nds29	Does your child give attention to common sounds?	Hearing
nds30	Do you always need to speak loudly to get the attention of your child?	Hearing
nds31	Does your child seem to have difficulty in comprehending what you are saying?	Intellectual Disability, Hearing
nds32	Does your child insist on sameness and actively resist any change in his/ her routines?	Autism
nds33	Does your child appear to be lost in his/her own world, no matter what he/she is doing?	Autism
nds34	Does the child do activities that are purposeless, repetitive and excessive	Autism
Nds35	Compared with other children of his /her age, does the mental/cognitive development of your child appear to be slow?	Intellectual Disability
nds36	Compared with other children of his /her age, does your child have difficulty in learning new things?	Autism, Intellectual Disability, Hearing, Visual, LD
nds37	As compared to other children of his/ her age is your child excessively active and he/she appears to be “on the go”?	ADHD
nds38	As compared to children of his/ her age, does your child appear to act, speak or behave without thinking?	ADHD
nds39	As compared to children of his/ her age, does your child have difficulty in sustaining attention on activities at school, home or play?	Intellectual Disability, ADHD

LD=learning disability; SLD=specific language disorders; ADHD=attention-deficit hyperactivity disorder

© 2025 Kariuki SM et al. *JAMA Network Open*.

eTable 2. Validity of all NDST questions in detecting neurodevelopmental disorders[1]

Impairment	Sensitivity (%) (95% CI)	Specificity (%) (95% CI)
ASD	96.5 (96.1-96.8)	80.6 (79.9-81.3)
ADHD	89.2 (88.7-89.8)	81.7 (81.0-82.4)
Epilepsy	88.8 (88.2-89.3)	80.6 (79.8-81.3)
Intellectual disability	88.2 (87.6-88.5)	80.3 (79.5-81.0)
Motor impairments	100.0 (100.0-100.0)	80.1 (79.3-80.8)
Visual impairments	100.0 (100.0-100.0)	80.0 (79.3-80.7)
Hearing impairments	85.7 (85.1-86.3)	80.0 (79.3-80.7)
All neurodevelopmental disorders	87.8 (87.3-88.5)	83.3 (82.7-84.0)

© 2025 Kariuki SM et al. *JAMA Network Open*.

eTable 3. Definitions of neurodevelopmental disorders

**Neurodevelopmental disorder**	**Definition/diagnosis criteria**
Epilepsy	A lifetime history two or more unprovoked seizures occurring 24 hours a part
Impaired Cognition as a marker of intellectual disability	Z-score of below -2 one on either of the neuropsychological tests administered i.e. Ravens Coloured Progressive Matrix Test or Kilifi Naming Test
Motor	Difficulty in holding implements, dressing and sitting upright. Able to move around with help. Inability to walk and absence of functional use of hands
Hearing	A 26-40 dB loss in the best ear and difficulty in hearing even with a hearing aid.
Vision	Vision loss of 6/18 m or worse.
Attention deficit hyperactivity disorder	Any child fulfilling a scoring system of the DSM-IV diagnostic criteria for K-SADS-PL, requiring presence of ADHD symptoms.
Autism spectrum disorder	Any child fulfilling a scoring algorithm of the DSM-IV diagnostic criteria for ADOS and 3Di, for ASD symptoms as directed by the developers of the tool

© 2025 Kariuki SM et al. *JAMA Network Open*.

eTable 4. Children with neurodevelopmental disorders according to age and sex

Neurodevelopmental disorders	Age								Total		p-value[1]
	6 years		7 years		8 years		9 years				
	Males N=821	Females N=830	Males N=1898	Females N=1905	Males N=1838	Females N=1798	Males N=1089	Females N=1044	Males N=5646	Females N=5577	
Attention deficit hyperactivity disorder (ADHD)	27 (2·8%)	13 (2·1%)	61 (3·2%)	38 (2·0%)	58 (3·2%)	40 (2·2%)	25 (2·3%)	23 (2·2%)	167 (3·0%)	118 (2·1%)	0·005
Autism spectrum disorder (ASD)	11 (1·3%)	7 (0·8%)	15 (0·8%)	18 (0·9%)	10 (0·5%)	13 (0·7%)	10 (0·9%)	12 (1·2%)	46 (0·8%)	50 (0·9%)	0·638
Cognitive impairment	19 (2·3%)	13 (1·6%)	25 (1·3%)	17 (0·9%)	20 (1·1%)	27 (1·5%)	18 (1·7%)	9 (0·9%)	82 (1·5%)	66 (1·2%)	0·212
Epilepsy	6 (0·7%)	4 (0·5%)	13 (0·7%)	16 (0·8%)	20 (1·1%)	16 (0·9%)	12 (1·1%)	11 (1·1%)	51 (0·9%)	47 (0·8%)	0·730
Motor impairments	2 (0·2%)	2 (0·2%)	2 (0·1%)	3 (0·2%)	1 (0·1%)	5 (0·3%)	0 (0·0%)	3 (0·3%)	5 (0·1%)	13 (0·2%)	0·056
Hearing impairments	2 (0·2%)	2 (0·2%)	4 (0·2%)	4 (0·2%)	0 (0·0%)	4 (0·2%)	2 (0·2%)	0 (0·0%)	8 (0·1%)	10 (0·2%)	0·619
Visual impairments	1 (0·1%)	1 (0·1%)	4 (0·2%)	3 (0·2%)	2 (0·1%)	1 (0·1%)	2 (0·2%)	0 (0·0%)	9 (0·2%)	5 (0·1%)	0·295
Any neurological impairments	17 (2·1%)	8 (1·0%)	26 (1·4%)	24 (1·3%)	26 (1·4%)	28 (1·6%)	19 (1·7%)	14 (1·3%)	88 (1·6%)	74 (1·3%)	0·303
Any developmental impairments	29 (3·5%)	23 (2·8%)	68 (3·6%)	52 (2·7%)	64 (3·5%)	49 (2·7%)	30 (2·8%)	29 (2·8%)	191 (3·4%)	153 (2·7%)	0·049
Any NDD	47 (5·7%)	37 (4·5%)	98 (5·2%)	80 (4·2%)	86 (4·7%)	80 (4·5%)	54 (5·0%)	40 (3·8%)	285 (5·1%)	237 (4·3%)	0·045

[1]Compares NDD cases between males and females using a Pearson's X^2 test, or Mann-Whitney U test where cases are infrequent.

© 2025 Kariuki SM et al. *JAMA Network Open*.

eTable 5. Risk factors for all neurodevelopmental disorders (NDDs) diagnosed in stage II

Risk factors	Without NDD (N=1,690)	With NDD (N=522)	Penultimate adjusted model: RR (95%CI)[1]	P-value	Multivariable model: RR (95%CI)[2]	P-value
**Pregnancy and birth information**						
Mental health problems in pregnancy	271/1608 (16.9%)	121/493 (24.5%)	1.36 (1.14-1.62)	0.001	1.18 (0.94-1.48)	0.146
Substance use/abuse (alcohol, smoking & caffeine) in pregnancy	139/1,608 (8.6%)	27/495 (5.5%)	0.64 (0.44-0.92)	0.016	0.68 (0.46-0.99)	0.047
Mother's age at first birth (years): median (IQR)	18.0 (18.0-20.0)	18.0 (17.0-20.0)	0.99 (0.97-1.02)	0.492	N/A	N/A
Pregnancy problems	245/1621 (15.1%)	113/504 (22.5%)	1.35 (1.13-1.62)	0.001	1.14 (0.90-1.44)	0.262
Delivery at home	1235/1625 (76.0%)	358/504 (71.0%)	0.83 (0.70-0.97)	0.027	0.89 (0.74-1.07)	0.207
Difficult delivery/birth	173/1608 (10.8%)	90/496 (18.2%)	1.48 (1.22-1.79)	<0.0001	1.11 (0.89-1.41)	0.379
Adverse perinatal events	87/1623 (5.4%)	73/504 (14.5%)	1.96 (1.62-2.37)	<0.0001	1.64 (1.29-2.09)	<0.0001
Neonatal jaundice	57/1596 (3.6%)	23/491 (4.7%)	1.13 (0.80-1.61)	0.483	N/A	N/A
Neonatal infections (tetanus & sepsis)	12/1608 (0.8%)	10/496 (2.0%)	1.76 (1.07-2.88)	0.025	1.27 (0.75-2.14)	0.375
Incomplete immunization	51/1585 (3.2%)	11/494 (2.2%)	0.73 (0.43-1.25)	0.252	N/A	N/A
Birthweight (Kg): median (IQR)	3.0 (2.6-3.5)	3.0 (2.7-3.5)	0.98 (0.76-1.25)	0.850	N/A	N/A
**Medical history information**						
Family history of seizures	311/1640 (19.0%)	115/510 (22.6%)	1.14 (0.95-1.36)	0.172	1.08 (0.89-1.31)	0.439
Previous hospital admission	390/1633 (23.9%)	179/506 (35.4%)	1.47 (1.26-1.72)	<0.0001	1.40 (1.18-1.66)	<0.0001
Acute febrile illness	202/1610 (12.6%)	97/498 (19.5%)	1.46 (1.21-1.76)	<0.0001	1.28 (1.05-1.56)	0.017
Head injury	82/1634 (5.0%)	45/507 (8.9%)	1.50 (1.17-1.92)	0.002	1.46 (1.13-1.88)	0.004
Eats cassava	1610/1643 (98.0%)	498/509 (98.0%)	0.91 (0.56-1.48)	0.706	N/A	N/A
Dogs or cats in compound	760/1645 (46.0%)	237/511 (46.4%)	1.02 (0.88-1.19)	0.754	N/A	N/A
Eats soil	92/1639 (5.6%)	55/508 (10.8%)	1.55 (1.24-1.94)	<0.0001	1.45 (1.13-1.85)	0.003
Snores at night	735/1635 (45.0%)	304/508 (60.0%)	1.54 (1.31-1.80)	<0.0001	1.50 (1.27-1.76)	<0.0001
Eats pork	343/1631 (21.0%)	107/507 (21.1%)	0.97 (0.81-1.18)	0.785	N/A	N/A
Bednet use	1551/1644 (94.3%)	492/508 (96.9%)	1.70 (1.08-2.66)	0.022	1.89 (1.09-3.24)	0.023
**Socioeconomic information**						

© 2025 Kariuki SM et al. *JAMA Network Open*.

Maternal factors						
Maternal age (years)	34 (29-40)	33 (28-39)	0.99 (0.98-1.00)	0.018	0.95 (0.97-1.00)	0.007
Mother is literate	1014/1622 (62.5)	312/503 (62.0%)	1.04 (0.88-1.22)	0.620	N/A	N/A
Mother is employed	1363/1619 (84.2%)	427/506 (84.4%)	1.03 (0.92-1.08)	0.792	N/A	N/A
Father is literate	1376/1556 (88.4%)	434/488 (88.9%)	1.08 (0.84-1.39)	0.539	N/A	N/A
Father is employed	1451/1569 (92.5%)	445/491 (90.6%)	0.89 (0.67-1.17)	0.387	N/A	N/A
Number of siblings: median (IQR)	5 (4-7)	5 (4-7)	0.98 (0.95-1.01)	0.288	N/A	N/A
Number of dead siblings: median (IQR)	0 (0-1)	0 (0-1)	1.07 (0.99-1.15)	0.075	1.11 (1.03-1.19)	0.006
Deceased parents	113/1629 (6.9%)	37/509 (7.3%)	0.94 (0.70-1.27)	0.689	N/A	**N/A**
House status						
Minor or no repairs	65/1640 (4.0%)	9/508 (1.8%)	1.00		1.00	
Incompletely built	267/1640 (16.3%)	84/508 (16.5%)	1.87 (0.99-3.50)	0.051	1.66 (0.80-2.98)	0.133
Major repairs needed	295/1640 (18.0%)	98/508 (19.3%)	2.05 (1.10-3.82)	0.024	1.88 (0.92-3.41)	0.059
Dilapidated house	1013/1640 (61.8%)	317/508 (62.4%)	1.90 (1.03-3.48)	0.039	1.60 (0.82-2.94)	0.148
Grass-thatch roof type	1099/1645 (66.8%)	358/511 (70.1%)	1.10 (0.93-1.29)	0.286	1.15 (0.95-1.38)	0.147
Land tenure						
Self-owned	1064/1637 (65.0%)	288/506 (56.8%)	**1.00[3]**		**1.00[3]**	
Rented/leased	45/1637 (2.8%)	13/506 (2.6%)	0.98 (0.59-1.64)	0.947	0.99 (0.58-1.67)	0.990
Landless/squatter	528/1637 (32.3%)	205/506 (40.7%)	1.25 (1.07-1.47)	0.005	1.32 (1.12-1.56)	0.001

[1]Adjusted for both child factors (age, sex, and schooling) and maternal factors (marital status, ethnicity and religion); [2]Variables reaching P≤0.25 in each sub-category were entered together into a multivariable model adjusted for the child and maternal factors for that subcategory; [3]the model showed statistically significant linear test for trend for the categories of these variable; N/A: Did not reach threshold for multivariable analysis; Risk factors were collected during stage II of the study

© 2025 Kariuki SM et al. *JAMA Network Open*.

eTable 6. Multivariable risk ratios (RRs) of factors associated with developmental conditions and with neurologic conditions

Risk factors	Developmental conditions (ASD or ADHD)		Neurological conditions	
	RR (95%CI)[1]	P-value	RR (95%CI)[1]	P-value
Substance use/abuse (alcohol, smoking & caffeine) in pregnancy	0.64 (0.39-1.07	0.088	0.73 (0.41-1.28)	0.272
Adverse perinatal events	1.99 (1.51-2.62)	<0.001	1.46 (1.01-1.09)	0.039
Previous hospital admission	1.21 (0.95-1.54)	0.116	1.58 (1.21-2.05)	0.001
Acute febrile illness	1.03 (0.77-1.38)	0.850	1.70 (1.27-2.27)	<0.001
Head injury	1.48 (1.03-2.12)	0.035	1.46 (0.94-2.27)	0.090
Eats soil	1.69 (1.23-2.33)	0.001	2.15 (1.52-3.03)	<0.001
Snores at night	1.60 (1.28-1.99)	<0.001	1.51 (1.16-1.95)	0.002
Bednet use	1.66 (0.76-3.64)	0.207	0.98 (0.49-1.93)	0.945
Maternal age (years)	0.99 (0.98-1.00)	0.402	0.98 (0.97-1.00)	0.117
Land tenure	1.24 (1.11-1.38)	<0.001	1.14 (1.00-1.29)	0.053
Number of dead siblings	1.07 (0.95-1.19)	0.251	1.17 (1.04-1.31)	0.007

ASD: Autism spectrum disorders: ADHD: Attention-Deficit Hyperactivity disorder; RR: risk ratio. [1]All risk factors were entered into one multivariable model

© 2025 Kariuki SM et al. *JAMA Network Open*.

eFigure. Overlap distribution by number of neurodevelopmental disorders

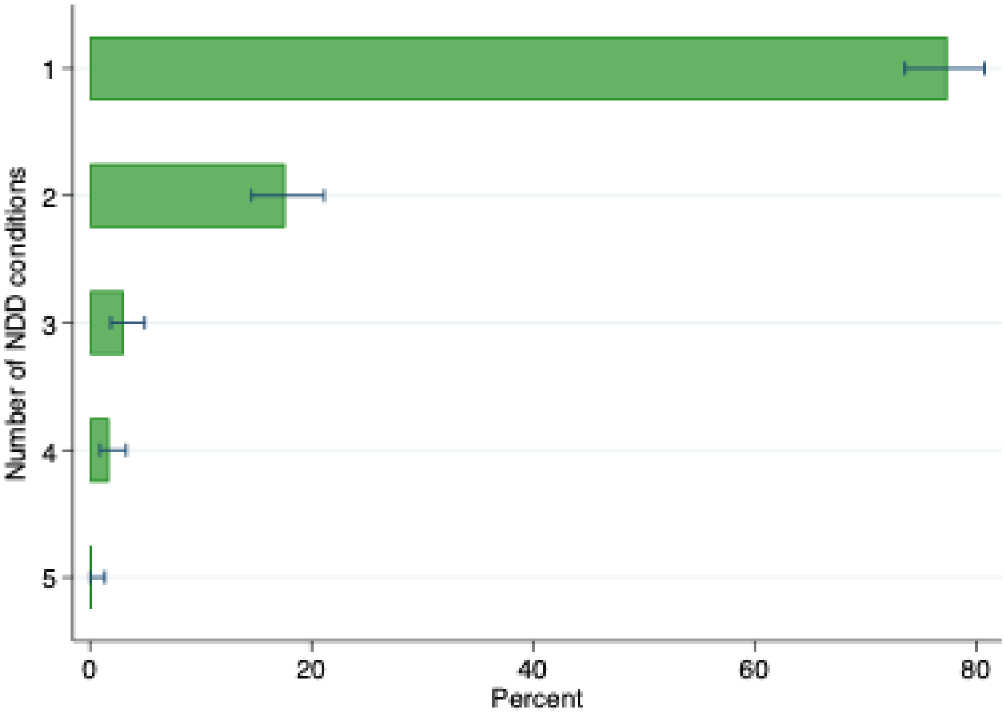

© 2025 Kariuki SM et al. *JAMA Network Open*.

eReference

1. Bitta MA, Kipkemoi P, Kariuki SM, et al. Validity and reliability of the Neurodevelopmental Screening Tool (NDST) in screening for neurodevelopmental disorders in children living in rural Kenyan coast. *Wellcome Open Res*. 2021;6:137. doi:10.12688/wellcomeopenres.16765.1

© 2025 Kariuki SM et al. *JAMA Network Open*.
