# Supplementary material for: Burden of Neurodevelopmental Disorders in Kenyan Children
Source: JAMA Netw Open. 2025 Dec 12;8(12):e2548853. doi: 10.1001/jamanetworkopen.2025.48853 (PMC12701508; doi:10.1001/jamanetworkopen.2025.48853)
Supplement: Supplement 2. — Data Sharing Statement [file jamanetwopen-e2548853-s002.pdf]

Data Sharing Statement

Kariuki. Burden of Neurodevelopmental Disorders in Kenyan Children. *JAMA Netw Open*. Published December 12, 2025. doi:10.1001/jamanetworkopen.2025.48853

Data
Data available: Yes
Data types: Deidentified participant data
How to access data: Data requests can be made through contacting: dgc@kemri-wellcome.org
When available: With publication

Supporting Documents
Document types: None

Additional Information
Who can access the data: Anyone with a proposed analysis plan will have access to the data.
Types of analyses: Any scientific analyses is eligible for data requests.
Mechanisms of data availability: Data will be accessible after approval of proposal.
Any additional restrictions: None
